# The uterine choriocarcinoma in postmenopausal women: specificities of diagnosis and treatment

**DOI:** 10.11604/pamj.2014.19.176.5403

**Published:** 2014-10-20

**Authors:** Ons Kaabia, Sawsen Meddeb, Mohamed Salah Rhim, Mohamed Bibi, Hedi Khairi

**Affiliations:** 1 Department of Gynecology Obstetrics, University Hospital Farhat Hached Sousse, Tunisia

**Keywords:** Uterine choriocarcinoma, post menopause, gestational trophoblastic tumor

## Abstract

Choriocarcinoma is a gestational trophoblastic tumor that mainly affects women of childbearing age. Cases of choriocarcinoma in postmenopausal women are exceptional. Through an observation and literature review, we propose to study the specific diagnosis and treatment features of this tumor in menopausal women. We report the observation of a pure uterine choriocarcinoma, which occurred in post-menopause. The diagnosis was made on the analysis of surgical specimens confirmed by measurement of hCG. Chemotherapy was started after a total hysterectomy and bilateral salpingo-oophorectomy first. The improvement was dramatic after 3 courses of chemotherapy and the patient is in complete remission after five years of monitoring. The primitive forms of pure choriocarcinoma in postmenopausal women are exceptional. Their etiology is poorly understood and their treatment based on chemotherapy.

## Introduction

Gestational Trophoblastic Tumors (GTT) are a rare complication of pregnancy and represent a wide spectrum of diseases containing the hydatiform mole (partial or total), the invasive mole, the choriocarcinoma and the tumor of the placental insertion site [1]. The choriocarcinoma is the most common malignant GTT. It is the most primitive, the less differentiated and the more chemo sensitive GTT [2]. Vascular invasion is standing [3]. It can complicate a previous mole or a normal pregnancy. This gestational tumor reaches preferentially women of childbearing age. Some morphs to post-menopausal revelation have been reported since 1970.

## Patient and observation

Ms J.A, 63 years, 7 living childre, menopaused for 14 years, is admitted for post-menopausal bleeding. During the examination, her conjunctiva was pale, the uterus was not felt in sus-pubic, and the vaginal touch was painless. The cervix was macroscopically healthy with blackish bleeding of low abundance. Pelvic ultrasound found an increased womb size (95 X 52 mm) and the thick endometrium contained a slightly hypoechogenous heterogeneous, richly vascularized peripheral myometrium to the Doppler tumor of the endometrium of 34 X 26 mm. There was no effusion in Douglas or adnexal abnormality. A malignant tumour of the endometrium was susected. A laparotomy with a total hysterectomy and bilateral salpingo-oophorectomy was initially performed after a negative extension staging. The pathology results of the operating pieces concluded an intrauterine choriocarcinoma partly infiltrating the myometrium without going beyond. The tumor is classified as stage I of FIGO, and low risk according to the WHO modified classification. Then, we completed a dosage of hCG on the preoperative blood sampling that came positive at 33652 IU/ml. The decision was to complete by 5 courses of chemotherapy according to the Protocol EPA (actinomycin d1, 2, 3 then d14 and 15, VP16 d1, 2, 3 then d14 and 15 and cisplatyl d1). Tolerance of chemotherapy was marked by a grade II neuropathy in the lower limbs,grade II alopecia, grade II vomiting, grade IV anemia and a grade IV neutropenia. The evolution was marked by the negativity of the rate of hCG after the 3th course of chemotherapy and the persistence of the negativity of the biological tests and of the neuropathy in the lower limbs after a 5 years follow up.

## Discussion

The incidence of choriocarcinoma and GTT varies geographically. The prevalence of GTT was estimated at 1 in 400 live births in the Arab countries and Asia by Rangwala et al. [4] and 1 per 1000 live births in developed countries [5]. The incidence was 12.1 per 1000 deliveries in Turkey between 1998 and 2003 with 6.9% of choriocarcinoma [6]. This tumor can develop in all women of childbearing age and especially beyond 40 years. In a Tunisian retrospective series, the average age was 32 years (range 20 to 49 years) [5]. The median time to onset of choriocarcinoma is 1 year after the last pregnancy [6]. Cases of choriocarcinoma were reported after menopause [7–9]. In 1985, Tsukamoto et al have published a series of 20 GTT in women aged 50 and over; 25% of which were choriocarcinoma, and all patients with amenorrhea were carriers of choriocarcinoma with amenorrhea of 11, 15 and 18. [9]. The larger historical series of GTT after 50 years, published by Jequier and Winterton, has identified 109 cases with malignant 28.4% in patients aged 51 to 64 years (54.2 years on average). Amenorrhea ranged from 0 to 22 years with a median of 12 months and an average of 33 months [10].

These choriocarcinoma diagnosed in postmenopausal women with gestational degeneration may be a molar pregnancy in its complete form (75%) or after a normal pregnancy carried to term, or after miscarriage, ectopic pregnancy [11, 12]. They may be primitive, not gestational, exceptional, sporadic, limited to the uterine level, almost always associated with ovarian damage [12, 13]. Their origin is explained by the degeneration of germ cells or somatic cells with malignant metaplasia of the epithelium under the influence of pre-existing oncogenes [3, 11, 12]. These primitive forms usually occur in older patients. They seem to be more aggressive with a poorer prognosis related to later diagnosis, explaining the frequent metastases. The study of DNA polymorphism can help to distinguish between gestational choriocarcinoma (presence of both paternal and maternal alleles in equal amounts in tumor cells) [11] and not gestational (maternal allele only) [11]. This method is expensive, time consuming and requires unfixed fresh tissue collected especially for this purpose [10]. In 20% of cases, previous pregnancy is not that at issue [14]. Note that generally, the more the period between the last pregnancy and diagnosis of the tumor is long, the more the prognosis is poor [14]. But in postmenopausal women, the prognostic value of this delay is uncertain.

The clinic of choriocarcinoma in postmenopausal women is not specific. Most patients consult for postmenopausal bleeding but some cases have arisen in a context of acute surgical abdomen [14]. Sometimes, the clinic may be dominated by secondary metastatic locations (especially lung but also brain, liver, digestive or urinary ones) [14]. The pelvic ultrasound images are not specific either: The tumor masses are relatively hypo-echogenic to the myometrium device with occasional millimeteric fluid beaches related to an identified richly vascularized glandulocystic aspect with a high diastolic flow and a low resistance index [15]. In case of uterine mass in postmenopausal women, it is relevant to retrace the obstetric history of the patient, especially the history of GTT or bleeding after a normal pregnancy or post-abortion. In this case, the dosage of hCG helped the diagnosis and during the follow up, yet it is not prescribed routinely in menopausal patients. The histological diagnosis of choriocarcinoma is quite reliable because when the first pathologist diagnosed achoriocarcinoma, the referent pathologist confirmed it in 86% of cases [14]. In cases of atypical morphology such as the predominance of cytotrophoblast and intermediate trophoblast which can form cohesive monomorphic cell ranges [9], there is a problem of differential diagnosis. It is where immunohistochemistry is the most helpful. If the hCG by immunolabeling is intense and diffuse, the diagnosis is highly possible [10]. The OCT-3/4, CD30 and AFP are not specific markers of germ cell tumors [15]. The OCT-3/4 is a transcription factor expressed in undifferentiated multipotent cells as germ cells. The CD30 is part of the TNF cytokines family and is used as a marker of embryonal carcinoma. The combined use of OCT-3/4 and CD30 helps establish the germ origin of metastatic tumors. If the marking is negative for both, the original germ may be excluded [15].

For uterine described forms, choriocarcinoma is never isolated, unlike our case report. It is found most often associated with adenocarcinomas but also with carcino-sarcomas or mixed mesodermal tumors [10]. Desai et al. reported the case of pure choriocarcinoma in a patient 73 years of menopause and the authors eliminated by curettage biopsy of the endometrium the hypothesis of dedifferentiation of endometrial carcinoma cells in the absence of carcinomatous component [5].

Choriocarcinoma is considered the most curable gynecologic cancer, even if metastatic and its overall survival rates are between 82-100% [10]. Chemotherapy has completely revolutionized the prognosis of these tumors with survival rates going from 19% to 90% (DR). Multidrug therapy is the gold standard first-line in metastatic forms of high-risk gestational tumors such as choriocarcinoma [13]. This typically includes: Etoposide, Methotrexate, Actinomycin D, Cyclophosphamide, and Vincristine [10]. The peculiarity of this poly chemotherapy, administered to older patients often with other chronic and severe illnesses, is the higher incidence of toxicity in the short, medium and long term. This is the case for our patient who keeps a lower extremity neuropathy. Other reported cases of postmenopausal choriocarcinoma with a significant morbidity and mortality of the chemotherapy: fatal outcome in a context of acute toxicity of chemotherapy (acute toxic epidermal lyses/ Leyell syndrome [16]); toxicity related to etoposide (malignant hypertension) requiring a switch of EMA-CO protocol to methotrexate with folic acid with negativity of hCG after 4 cycles and incident-free survival of 20 months [16]. The toxic death rate of 28% with EPA protocol is unacceptable in the context of a curable disease in more than 80% of cases. The AE protocol, less toxic, was still responsible for a frequent gastrointestinal toxicity (87%) and dermatological side effects in 25% of cases in a retrospective study of Tunisia [8].

The surgery is performed, generally, in a second phase, due to the association with other histologic carcinoma. If the diagnosis is based on the hysterectomy specimen, chemotherapy will depend on the evolution of hCG and staging. This raises the possibility of first non-conservative surgery in these menopausal patients by total hysterectomy associated with bilateral salpingo-oophorectomy. Surgery is usually indicated as first-line in case of a persistent trophoblastic disease in women at high risk with no desire of pregnancy, or for hemostatic goal [15]. The main advantage of the first hysterectomy followed by chemotherapy versus chemotherapy alone is to lower total doses of drugs [6]. This applies mainly to etoposide, known for its acute toxicity and for its long-term risks of chemotherapy-induced neoplasms, both of which are correlated with cumulative doses received by patients. Surgery of residual masses is unnecessary as the hCG is negative [16].

## Conclusion

This case and literature review illustrate that the diagnosis of choriocarcinoma is possible and must be mentioned even in post-menopausal endometrial before any image and heterogeneous, thus, think of the hCG assay, simple and inexpensive examination. This tumor chemo-sensitive or even curable metastatic has a prognosis that depends mainly on early diagnosis and therefore treatment. Knowing that multidrug therapy can itself be a source of significant morbidity or mortality, it should be strictly monitored.

## References

[1] El-Helw LM, Hancock BW (2007). Treatment of metastatic gestational trophoblastic neoplasia. Lancet Oncol..

[2] Shih LeM (2007). Gestational trophoblastic neoplasia-pathogenesis and potential therapeutic targets. Lancet Oncol..

[3] Le Bret T, Tranbaloc P, Benbunan J-L, Salet-Lizée D, Villet R (2005). Choriocarcinome utérin en péri-ménopause. J Gynecol Obstet Biol Reprod (Paris).

[4] Rangwala TH, Badawi F (2011). Profile of Cases of Gestational Trophoblastic Neoplasia at a Large Tertiary Centre in Dubai. ISRN Obstetrics and Gynecology..

[5] Desai NR, Gupta S, Said R, Desai P, Dai Q (2010). Choriocarcinoma in a 73-year-old woman: a case report and review of the literature. J Med Case Rep..

[6] Harma M, Yurtseven S, Gungen N (2005). Gestational trophoblastic disease in Sanliurfa, Southeast Anatolia, turkey. European Journal of Gynaecologic Oncology..

[7] O'Neill CJ, Houghton F, Clarke J, McCluggage WG (2008). Uterine gestational choriocarcinoma developing after a long latent period in a postmenopausal woman: the value of DNA polymorphism studies. Int J Surg Pathol.

[8] Khanfir A, Masmoudi A, Toumi N, Slimi Kallel L, Guermazi M, Boudawara T, Frikha M (2010). Maladies trophoblastiques gestationnelles persistantes: une étude de 26 cas. J Afr Cancer..

[9] Marcu M, Chefani A, Sajin M (2005). Postmenopausal choriocarcinoma: a case report. Rom J Morphol Embryol..

[10] Jequier A, Winterton WR (1973). Diagnostic problems of trophoblastic disease in women aged 50 or more. Obstet Gynecol..

[11] Dilek S, Pata O, Tok E, Polat A (2004). Extra ovarian non gestational choriocarcinoma in a postmenopausal woman. Int J Gynecol Cancer..

[12] Fisher RA, Savage PM, MacDermott C, Hook J, Sebire NJ, Lindsay I, Seckl MJ (2007). The impact of molecular genetic diagnosis on the management of women with hCG-producing malignancies. Gynecol Oncol..

[13] Mukherjee U, Thakur V, Katiyar D, Goyal HK, Pendharkar D (2006). Uterine choriocarcinoma in a postmenopausal woman. Med Oncol..

[14] Genest DR, Berkowitz RS, Fisher RA, Newlands ES, Fehr M, Tavassoli Fattaneh A (2003). Gestational trophoblatic disease. Pathology and Genetics of Tumours of the Breast and Female Genital Organs: World Health Organization Classification of Tumours.

[15] Ulbright TM (2005). Germ cell tumors of the gonads: a selective review emphasizing problems in differential diagnosis, newly appreciated, and controversial issues. Mod Pathol..

[16] Chittenden B, Ahamed E, Maheshwari A (2009). Choriocarcinoma in a postmenopausal woman. Obstet Gynecol..

